# Who drinks sugar sweetened beverages and juice? An Australian population study of behaviour, awareness and attitudes

**DOI:** 10.1186/s40608-018-0224-2

**Published:** 2019-01-03

**Authors:** Caroline Miller, Melanie Wakefield, Annette Braunack-Mayer, David Roder, Kerin O’Dea, Kerry Ettridge, Joanne Dono

**Affiliations:** 1 School of Public Health, University of Adelaide, Adelaide, Australia; 2 South Australian Health and Medical Research Institute (SAHMRI), Population Health Research Group, North Terrace, Adelaide, South Australia Australia; 3 Cancer Council Victoria, Centre for Behavioural Research in Cancer, Melbourne, Australia; 4 School of Health & Society, University of Wollongong, Wollongong, New South Wales Australia; 5 Centre for Population Health Research, University of South Australia, Adelaide, Australia

**Keywords:** Sugar-sweetened beverages, 100% fruit juice, Population survey, Risk factors, Attitudes, Knowledge, Awareness

## Abstract

**Background:**

The rate of overweight and obesity in Australia is among the highest in the world. Yet Australia lags other countries in developing comprehensive educative or regulatory responses to address sugary drink consumption, a key modifiable risk factor that contributes substantial excess sugar to the diet. Measurement of sugary drink consumption is typically sporadic and nutrition focussed and there is limited knowledge of community perceptions and awareness of the health risks associated with excess sugary drink consumption. The aim of this study was to assess the demographic characteristics, behavioural risk factors and attitudes and knowledge associated with sugar-sweetened beverage (SSB) and 100% fruit juice consumption.

**Methods:**

A face-to-face household survey was conducted in 2014 using a stratified random sampling strategy to represent the South Australian population aged 15 years and over. The survey contained questions on sugary drinks, with past week SSB consumption and 100% fruit juice consumption used as outcome variables. Associations were examined with demographic characteristics, behavioural risk factors, and sugary drink attitudes and knowledge.

**Results:**

Of the 2732 respondents, 35% had consumed SSBs 1–6 times (moderate consumers) and 16% had consumed SSBs 7 or more times (frequent consumers) in the past week. Furthermore, 35% had consumed 100% fruit juice in the past week, with 10% consuming every day. Rates of SSB consumption were consistently higher among males, younger age groups, and groups with lower education attainment, as well as smokers and frequent consumers of fast food. Awareness of health risks and sugar content of SSBs was low, especially among frequent SSB consumers. Fruit juice consumption was higher among males, younger age groups, the physically active and among those believing that 100% fruit juice did not contain more sugar than SSBs.

**Conclusions:**

Consumption of SSBs and 100% fruit juice is common but awareness of health risks and sugar content of these drinks is low. There is a need for greater consumer understanding which could be achieved through educative approaches such as public education campaigns, on-package warning labels and improved nutrition information panels.

**Electronic supplementary material:**

The online version of this article (10.1186/s40608-018-0224-2) contains supplementary material, which is available to authorized users.

## Background

Excess consumption of added and free sugars are gaining increasing attention as an environmental driver of obesity [1]. Within this context, sugar-sweetened beverages (SSBs) are a focus due to their energy density, coupled with poor nutritional value, and the strength of evidence linking their consumption with weight gain, obesity [2], Type 2 diabetes [3], tooth decay [4] and emergent evidence of cardiovascular risks [5]. Countries are moving to try to reduce their population consumption of SSBs and a raft of educative and regulatory interventions are being implemented [6, 7]. Australia lags other countries in comprehensive educative or regulatory responses to address SSB consumption and obesity more broadly [8].

At 63%, the rate of overweight and obesity in Australia is among the highest in the world [9], with the rate of obese Australians tripling since 1990. Australians are also high consumers of SSBs [10], and SSBs contribute substantial excess sugar to the national diet. Over half of Australians exceed the World Health Organization (WHO) recommendations for free sugar in the diet, with 52% of free sugars coming from beverages, notably soft drinks (sodas), electrolyte (sports) and energy drinks (19%), as well as fruit and vegetable juices and drinks (13%) [10].

To date, detailed monitoring of SSB consumption patterns has been infrequent and protracted due to the complexity of population-level dietary surveys. Consequently, it has offered limited insight into the behavioural and attitudinal correlates of SSB consumption. Australian national data collection last occurred in 2011–12, indicating that 50% of Australians consumed an SSB on the day before the interview [11]. Rates of consuming 100% fruit juice were lower at 23% for children (2–18 years) and 15% for adults (19 years and over) with few demographic differences [12]. Rates of SSB consumption were higher among males compared to females, and for adolescents and young adults compared to other age groups [11]. Another study reporting on state-based data collected in 2009 (Western Australia) and 2012 (South Australia) indicated that SSB consumers were more likely to be male, have little interest in health, or have purchased meals away from home [13]. Other research has demonstrated that frequent SSB consumption is associated with other poorer dietary consumption patterns, including regular fast food consumption [14–17].

Measurement of sugary drink consumption is typically sporadic and nutrition focussed, and there is limited knowledge of community perceptions and awareness of the health risks associated with excess SSB consumption. The current study sought to fill this gap by generating essential population-based evidence to inform public health efforts to reduce consumption. A key aim of the study was to determine the frequency of past week SSB consumption and examine the correlates of consumption. SSB consumption was defined as frequency of past week consumption of any of the following: soft drinks; energy drinks; sports drinks; fruit drinks or cordials; and excluded 100% fruit juice and artificially sweetened drinks. The SSB definition excluded 100% fruit juice, which, although somewhat controversial (e.g. Rampersaud et al. [18]) is increasingly acknowledged as a problematic source of free sugar and excess calories (e.g. Popkin & Hawkes [19]). A second unique aim of the study was to explore the prevalence and correlates of 100% fruit juice consumption.

## Methods

The South Australian Health Omnibus Survey (SAHOS) was used to collect data. The survey utilised a multi-stage, stratified, random sampling strategy to identify households eligible for inclusion. The sampling frame represented the South Australian population aged 15 years and over residing in areas with 1000 people or more. One interview was conducted per household, with the person whose birthday occurred last selected for interview. Up to six call back visits were made to obtain the interview of the eligible selected person. Participants were interviewed face-to-face by trained research assistants. An approach letter was sent 2 weeks in advance of the interview. The letter contained the study aims, ethics committee contact information, and details about participation, including that it was voluntary and results would be anonymous. Verbal agreement to participate in the study was considered informed consent and explicit verbal consent was obtained from parents/guardians for participants aged 15 to 17 years. Pilot testing occurred in August and field-work for the full study occurred between September and December 2014. From the 5200 households selected, 2732 interviews were conducted, yielding a response rate (i.e. proportion of completed interviews from initial eligible sample) of 54.5% and a participation rate (i.e. proportion of completed interviews from initial eligible sample where contact was established) of 60.6%. The study, including the approach to informed consent, was approved by the University of Adelaide Human Research Ethics Committee.

The SAHOS contained approximately 150 health and socio-demographic related questions requiring self-reported responses. This study reports on responses to a subset of questions pertaining to correlates of SSB consumption. The wording of questions, including definitions, are reported in Additional file 1 along with the corresponding variable sub-categories used in the analysis. For the first set of the analyses, SSB consumption was the outcome variable. SSBs were defined as all non-alcoholic water-based beverages with added sugar, including soft drinks, energy drinks, fruit drinks, sports drinks and cordials. The definition excluded milk-based products, 100% fruit juice or artificially sweetened beverages. SSB consumption was calculated by multiplying two questions: ‘number of days consumed SSBs in past week’ and ‘frequency of consumption per day’. Responses were split into categories: ‘none’ vs ‘any’ (1 or more drinks per week). As daily consumption is often reported in studies using dietary interviews (e.g. 11), ‘any’ consumption was split into ‘moderate’ (1 to 6 drinks) and ‘frequent’ (7 or more drinks) to approximate levels of consumption equivalent to less than daily versus daily, respectively. Predictor variables were grouped into three categories: demographic characteristics (gender, age, highest qualification and postcode derived socio-economic disadvantage [20] and remoteness [21]); risk factors (Body Mass Index [BMI; calculated from self-reported height and weight], past week physical activity, fast food consumption, 100% fruit juice consumption and smoking status); and SSB attitudes and knowledge (teaspoons of sugar in can of soft drink, perceived healthiness of diet soft drinks compared to SSBs, beliefs about sugar content of 100% fruit juice compared to SSBs, and knowledge of illnesses related to SSB consumption). The association between 100% fruit juice consumption, defined as having ‘none’ or ‘any’ (1 or more in the past week), and demographic characteristics and risk factors were also explored.

Statistical analyses were conducted using SPSS version 24 [22]. Descriptive analyses of the association between participant characteristics and 1) SSB consumption (none, moderate or frequent) and 2) 100% fruit juice consumption (none or any) were undertaken using Pearson’s chi-square tests. The adjusted standardised residual for each cell of the Pearson’s chi-square test was used to detect whether the obtained value for each demographic subgroup was lower or higher than expected relative to the percentages for overall SSB consumption. The Mantel-Haenszel test of linear trends was also used for the SSB outcome variable. Multivariate analyses were used to test the same relationships while also controlling for the influence of other variables. The ‘Complex samples: Logistic regression’ analysis in SPSS was used to control for the clustered sampling design frame. Demographic characteristics were analysed as a group of predictors for both SSB and 100% fruit juice consumption. Subsequent analyses controlled for demographic characteristics while testing the association between SSB consumption and 1) risk factors and 2) SSB attitudes and knowledge; and between 100% fruit juice consumption and risk factors. Data were weighted by the inverse of the individual’s probability of selection, as well as the response rate in metropolitan and country regions and then re-weighted to benchmarks derived from the June 2013 ABS Estimated Resident Population [23].

## Results

Just over half of respondents had consumed SSBs at least once in the past week, either 1 to 6 times (i.e., moderate consumption; 35%) or 7 or more times (i.e. frequent consumption; 16%). Just over a third of respondents had consumed 100% fruit juice either 1 to 6 days (25%) or every day (10%) in the past week. Overall 19.7% had consumed both 100% fruit juice and SSBs in the past week, whereas 33.8% had consumed neither 100% fruit juice nor SSBs.

Demographic, BMI and behavioural risk factors and attitude and knowledge characteristics of the 2732 respondents included in the study are displayed in Table 1. SSB consumption was significantly associated with nearly all the variables listed in Table 1. Many of the relationships exhibited a linear trend with each categorical increase in consumption. Moderate and frequent consumers shared similar characteristics, and the most pronounced differences were between frequent consumers and non-consumers. Based on the adjusted standardised residuals of the Pearson chi-square test, frequent consumers were more likely than non-consumers to be male compared to female, younger (15–24 years) compared to older (45–64 years) participants, have lower compared to higher education, live in areas of higher disadvantage compared to low disadvantage, and live in remote compared to metropolitan areas. The highest rates of frequent SSB consumption in the past week were among those consuming fast food two or more times in the past week (42%) and current smokers (38%). Consumption of 100% fruit juice was more likely among moderate SSB consumers than non-consumers. Physical activity had a non-linear trend with SSB consumption group; frequent consumers were less likely to be physically active, moderate consumers were more likely to participate in some activity, and non-consumers were more likely to be the most active. There was no association between consumption and self-reported Body Mass Index.

**Table 1:** Respondent characteristics and sugar sweetened beverage (SSB) consumption by demographic subgroup (*N* = 2372)

	Overall sample	SSB consumption in past week by demographic subgroup^a^							Chi-square tests		
			None		Moderate (1–6 times)		Frequent (7+ times)			Pearson	Trend^f^
	%	N	%		%		%		N	*P*-value	P-value
SSB consumption in past week^e^	100.0	2732	48.8		34.7		16.0				
Demographics											
Gender									2719	< 0.001	< 0.001
Male	49.2	1337	38.4	↓	40.8	↑	20.8	↑			
Female	50.8	1382	59.3	↑	29.2	↓	11.4	↓			
Age (years)									2717	< 0.001	< 0.001
15–24	16.0	430	27.4	↓	50.0	↑	22.6	↑			
25–44	32.1	872	38.6	↓	39.8	↑	21.6	↑			
45–64	31.6	861	54.5	↑	32.9		12.7	↓			
65 and over	20.3	554	73.8	↑	18.8	↓	7.4	↓			
Highest qualification^b^									2716	< 0.001	< 0.001
High School or less	39.4	1069	45.0	↓	35.3		19.7	↑			
Vocational	35.8	977	47.5		34.7		17.8				
University	24.7	670	57.6	↑	34.8		7.6	↓			
Disadvantage quintile									2719	< 0.001	< 0.001
Quintile 1 (most disadvantaged)	23.2	628	46.2		32.2		21.7	↑			
Quintile 2	16.2	441	44.7	↓	34.9		20.4	↑			
Quintile 3	20.1	548	47.6		35.8		16.6				
Quintile 4	21.1	577	50.8		39.3	↑	9.9	↓			
Quintile 5 (least disadvantaged)	19.3	525	55.8	↑	32.4		11.8	↓			
Remoteness									2721	< 0.001	< 0.001
Metropolitan	74.8	2034	49.9		35.9		14.2	↓			
Inner Regional	9.5	259	48.3		37.5		14.3				
Outer Regional	13.4	366	47.0		27.9	↓	25.1	↑			
Remote/very remote	2.3	62	35.5	↓	32.3		32.3	↑			
Body Mass Index^d^									2710	0.719	0.494
Underweight or healthy	38.6	1048	49.0		35.3		15.6				
Overweight	29.9	814	47.5		35.9		16.6				
Obese	21.0	572	52.1		32.2		15.7				
Don’t know either height or weight	10.1	276	47.1		35.9		17.0				
Behavioural risk factors											
Physical activity (past week)^c^									2718	< 0.001	0.071
None	18.7	509	47.5		32.0		20.4	↑			
1 to 6 days	58.0	1578	47.7		38.7	↑	13.7	↓			
Everyday	23.1	631	53.7	↑	27.9	↓	18.4				
Fast food consumption (past week)^b^									2718	< 0.001	< 0.001
None	52.4	1430	64.3	↑	28.0	↓	7.7	↓			
Once	29.2	790	42.2	↓	43.0	↑	14.8				
Two or more times	18.3	498	16.3	↓	41.8	↑	42.0	↑			
100% fruit juice consumption (past week)^d^									2712	< 0.001	0.007
None	64.7	1764	52.3	↑	31.3	↓	16.4				
One or more times	35.0	948	43.2	↓	41.5	↑	15.3				
Smoking status									2718	< 0.001	< 0.001
Current smoker	15.3	417	30.7	↓	31.2		38.1	↑			
Ex-smoker	28.9	789	56.9	↑	29.8	↓	13.3	↓			
Never smoked	55.7	1512	50.0		38.6	↑	11.4	↓			
Attitudes and knowledge											
Teaspoons of sugar in can of soft drink^c^									2713	< 0.001	0.093
Underestimate 0 to 7	29.8	809	42.4	↓	38.6	↑	19.0	↑			
Approx correct 8 to 12^g^	33.5	910	48.6		37.1		14.3				
Overestimate 13 to 99	20.9	568	52.5		35.7		11.8	↓			
Don’t know	15.6	426	58.0	↑	22.5	↓	19.5	↑			
Diet soft drinks versus SSBs^b^									2718	0.002	0.436
More healthy	17.3	473	52.9		33.0		14.2				
Less healthy	26.8	727	44.4	↓	35.2		20.4	↑			
The same	50.8	1384	49.3		36.1		14.6	↓			
Don’t know	4.9	134	57.5	↑	28.4		14.2				
100% fruit juice versus SSBs^b^									2717	< 0.001	< 0.001
More sugar	8.6	234	47.0		31.6		21.4	↑			
Less sugar	40.8	1111	45.4	↓	36.3		18.4	↑			
The same^g^	42.5	1156	52.2	↑	35.2		12.6	↓			
Don’t know	8.1	216	53.7		29.6		16.7				
Awareness of illnesses/health effects related to SSB consumption									2719	< 0.001	< 0.001
Weight gain											
No	57.5	1566	45.7	↓	36.7	↑	17.7	↑			
Yes^g^	42.5	1153	53.7	↑	32.5	↓	13.8	↓			
Diabetes									2719	< 0.001	< 0.001
No	39.0	1061	44.8	↓	34.0		21.2	↑			
Yes^g^	61.0	1658	51.8	↑	35.5		12.7	↓			
Tooth decay									2719	0.601	0.761
No	70.9	1933	49.5		34.4		16.2				
Yes^g^	29.1	786	48.0		36.4		15.6				
Heart disease									2718	0.022	0.036
No	85.1	2314	48.6		34.5		16.9	↑			
Yes	14.9	404	51.5		37.1		11.4	↓			

*Note*: Adjusted standardised residuals used to detect statistical significance within cells of Pearson’s chi-square results (represented as arrows); Relative to percentages for overall SSB consumption in the past week, cells with percentages greater than expected = ↑ and cells with values lower than expected = ↓ at the *p* < 0.05 level

^a^Excluding ‘not stated’ response category; ^b^Not stated = 0.1%, ^c^not stated = 0.2%, ^d^not stated = 0.3%, ^e^not stated = 0.5%

^f^Mantel-Haenszel test of linear trends; ^g^Most correct answer based on current evidence

Differences in attitudes and knowledge between consumption subgroups were also greatest between frequent consumers and non-consumers, although trends were not always linear. Overall, 34% of participants gave a response approximating the correct number of teaspoons of sugar (8 to 12) in a 375 ml (12.7 oz) can of soft drink (soda). Underestimating sugar content in soft drink was more common in moderate and frequent consumers than in non-consumers. Diet soft drinks (soda) and SSBs were rated as having the same level of healthiness by 51% of participants whereas 27% rated diet soft drinks as less healthy. Frequent consumers of SSBs were more likely to rate diet soft drinks as less healthy than the same level of healthiness. Equivalent proportions of participants accurately believed that 100% fruit juice contained the same amount of sugar as SSBs (43%) or believed juice had less (41%). Compared to non-consumers, frequent SSB consumers were less likely to rate 100% fruit juice as having the same amount of sugar as SSBs, but were more likely to rate it as having either more sugar or less sugar. Unprompted awareness of illnesses known to be associated with SSB consumption ranged from 15% for heart disease risk to 61% for diabetes. Awareness of illnesses/health effects (weight gain, diabetes and heart disease) was negatively associated with consumption.

Table 2 displays logistic regression results that tested the association between ‘none’ versus ‘any’ SSB consumption and demographic characteristics, BMI and behavioural risk factors and attitudes and knowledge. The odds of being a SSB consumer was consistently greater for males compared to females, for all age groups under 65 years compared to over 65 years, and was greatest for those aged 15 to 24 years, for those with vocational qualifications or less compared to university qualifications, and for those living in remote/very remote areas compared to metropolitan areas. Risk factors associated with consumption, controlling for demographics, were fast food consumption, 100% fruit juice consumption and smoking status. The association between SSB consumption and consuming fast food two or more times in the past week (compared to none) was particularly strong at over 5 times the odds. There were few statistically significant relationships between attitudes and knowledge and consumption when controlling for demographics. The odds of being a consumer were slightly greater for those who rated diet soft drink as less healthy than SSBs compared to those who rated them as healthier, and for those who did not recall weight gain as being related to consumption compared to those who did.

**Table 2:** Logistic regression of ‘any’ versus ‘none’ past week sugar sweetened beverage (SSB) consumption

	1. Demographics			2. Demographics & risk factors			3. Demographics & knowledge		
	OR	95% CI		OR	95% CI		OR	95% CI	
		Lower	Upper		Lower	Upper		Lower	Upper
(N)	2714			2696			2705		
Demographics									
Gender									
Male	2.5***	2.0	3.1	2.1***	1.7	2.6	2.4***	1.9	2.9
Female	1			1			1		
Age (years)									
15–24	7.6***	5.3	10.8	4.3***	2.9	6.5	7.4***	4.9	11.2
25–44	5.5***	4.2	7.1	3.3***	2.5	4.4	5.7***	4.3	7.4
45–64	2.5***	1.9	3.3	1.9***	1.5	2.5	2.6***	2.0	3.5
65 and over	1			1			1		
Highest qualification									
High School or less	2.0***	1.5	2.6	1.7***	1.4	2.2	1.9***	1.5	2.4
Vocational	1.7***	1.3	2.2	1.6**	1.2	2.1	1.6***	1.3	2.1
University	1			1			1		
Disadvantage quintile									
Quintile 1 (most disadvantaged)	1.4	1.0	1.9	1.1	0.8	1.6	1.3	0.9	1.9
Quintile 2	1.4	1.0	1.9	1.1	0.8	1.6	1.2	0.9	1.8
Quintile 3	1.4	1.0	1.9	1.2	0.9	1.7	1.3	0.9	1.9
Quintile 4	1.2	0.9	1.6	1.0	0.8	1.4	1.1	0.8	1.5
Quintile 5 (least disadvantaged)	1			1			1		
Remoteness									
Metropolitan	1			1			1		
Inner Regional	1.0	0.7	1.6	1.1	0.7	1.7	1.1	0.7	1.6
Outer Regional	1.0	0.8	1.4	1.0	0.8	1.2	1.0	0.8	1.4
Remote/very remote	1.7**	1.3	2.4	1.4*	1.1	1.9	1.8***	1.4	2.4
BMI and Behavioural risk factors									
Body Mass Index (BMI)									
Underweight or healthy				1					
Overweight				1.2	0.9	1.5			
Obese				1.0	0.8	1.2			
Don’t know height or weight				0.9	0.6	1.4			
Physical activity (past week)									
None				1					
1 to 6 days				1.0	0.8	1.2			
Everyday				0.8	0.5	1.1			
Fast food consumption (past week)									
None				1					
Once				1.9***	1.6	2.4			
Two or more times				5.3***	3.5	8.0			
100% fruit juice consumption (past week)									
None				1					
One or more times				1.3*	1.0	1.7			
Smoking status									
Current smoker				1.7**	1.2	2.5			
Ex-smoker				0.9	0.7	1.1			
Never smoked				1					
Attitudes and knowledge									
Teaspoons of sugar in can of soft drink									
Approx correct 8 to 12							1		
Underestimate 0 to 7							1.2	1.0	1.6
Overestimate 13 to 99							0.8	0.6	1.0
Don’t know							0.8	0.6	1.1
Diet soft drinks versus SSBs									
More healthy							1		
Less healthy							1.3*	1.0	1.8
The same							1.1	0.8	1.4
Don’t know							1.1	0.7	1.8
100% Fruit juice versus SSBs									
More sugar							1		
Less sugar							1.1	0.8	1.5
The same							0.9	0.7	1.3
Don’t know							0.8	0.5	1.4
Awareness of illnesses/health effects related to SSB consumption									
Weight gain (ref = Recalled)							1		
Not recalled							1.2*	1.0	1.4
Diabetes (ref = Recalled)							1		
Not recalled							1.1	1.0	1.3
Tooth decay (ref = Recalled)							1		
Not recalled							1.0	0.8	1.3
Heart disease (ref = Recalled)							1		
Not recalled							1.0	0.8	1.3

Logistic regression outcome variable: Any SSB consumption in past week = 1, none = 0

***p < 0.001; **p < 0.01; *p < 0.05

As shown in Table 3, 35% of respondents reported consuming 1 or more 100% fruit juice drinks in the past week. There were bi-variate associations between 100% fruit juice consumption and all the demographics and risk factor variables listed in Table 3 except for self-reported Body Mass Index. In the logistic regression testing demographic characteristics only (not reported in table), 100% fruit juice consumption in the past week was only associated with gender (males more likely than females; OR = 1.5, 95%CI = 1.2–1.8, *p* < 0.001) and age (15–24 years [OR = 1.4, 95%CI = 1.1–1.9, *p* = 0.005] and 25–44 years [OR = 1.4, 95%CI = 1.1–1.97, p = 0.005] more likely than those aged 65 years and over). In the combined demographic and risk factor model (see Table 3), past week 100% fruit juice consumption was more likely among males compared to females, those who participated in physical activity everyday compared to none in the past week, and those who rated 100% fruit juice as having the same or less sugar as SSBs rather than more sugar. There was less likelihood of consuming 100% fruit juice among ex-smokers compared to those who had never smoked.

**Table 3:** Association between 100% fruit juice consumption and respondent characteristics (*N* = 2732)

	100% fruit juice consumption in past week			Logistic regression		
	None	1 or more	Pearson χ^2^	OR	95% CI	
	%	%	P-value	(*N* = 2702)	Lower	Upper
100% fruit juice consumption in past week^c^	64.7	35.0				
Demographics						
Gender			< 0.001			
Male	60.4	39.6		1.5***	1.2	1.8
Female	69.2	30.8				
Age (years)			0.001			
15–24	60.5	39.5		1.2	0.9	1.5
25–44	61.3	38.7		1.2	0.9	1.5
45–64	67.7	32.3		1.0	0.8	1.2
65 and over	69.5	30.5		1		
Highest qualification^a^			0.017			
High School or less	66.5	33.5		0.9	0.6	1.2
Vocational	66.2	33.8		0.8	0.7	1.0
University	60.3	39.7		1		
Disadvantage quintile			0.002			
Quintile 1 (most disadvantaged)	70.8	29.2		0.8	0.6	1.2
Quintile 2	65.4	34.6		1.0	0.7	1.4
Quintile 3	63.6	36.4		1.1	0.8	1.5
Quintile 4	60.0	40.0		1.3	0.9	1.7
Quintile 5 (least disadvantaged)	64.1	35.9		1		
Remoteness			0.006			
Metropolitan	63.4	36.6		1		
Inner Regional	64.1	35.9		1.0	0.6	1.5
Outer Regional	72.9	27.1		0.7	0.5	1.1
Remote/very remote	67.7	32.3		0.9	0.6	1.3
BMI and behavioural risk factors						
Body Mass Index (BMI)^c^			0.205			
Underweight or healthy	64.5	35.5		1		
Overweight	62.9	37.1		1.2	0.9	1.5
Obese	68.4	31.6		1.1	0.8	1.3
Don’t know height or weight	65.2	34.8		1.1	0.7	1.8
Physical activity (past week)^b^			< 0.001			
None	73.0	27.0		1		
1 to 6 days	64.2	35.8		1.3	1.0	1.9
Everyday	60.6	39.4		1.8***	1.3	2.5
Fast food consumption (past week) ^a^			0.006			
None	67.6	32.4		1		
Once	61.2	38.8		1.2	1.0	1.5
Two or more times	62.8	37.2		1.1	0.9	1.5
Smoking status			< 0.001			
Current smoker	64.5	35.5		0.9	0.7	1.2
Ex-smoker	71.7	28.3		0.6***	0.5	0.8
Never smoked	61.4	38.6		1		
100% fruit juice versus SSBs^a^			< 0.001			
More sugar	75.3	24.7		1		
Less sugar	60.9	39.1		2.1***	1.6	2.9
The same	65.2	34.8		1.7**	1.2	2.5
Don’t know	72.1	27.9		1.4	0.8	2.3

Logistic regression outcome variable: Any 100% fruit juice consumption in past week = 1, none = 0

^a^Not stated = 0.1%, ^b^not stated = 0.2%, ^c^not stated = 0.3%

***p < 0.001; **p < 0.01; *p < 0.05

## Discussion

Using our brief measure, more than half of the participants in this study had consumed SSBs in the past week, with 16% consuming SSBs frequently (7 or more drinks weekly). Over one third of respondents had consumed 100% fruit juice in the past week, with 10% consuming every day. Consistent with other Australian data [10, 13, 24], consumption of SSBs in the past week was consistently higher among males, younger age groups and groups with lower educational attainment. Similarly, 100% fruit juice consumption was higher among males (in both bivariate and multivariate comparisons), and among younger age groups in bivariate (unadjusted) analyses. Unlike SSB consumption, 100% fruit juice consumption was higher among those with higher educational attainment and among less disadvantaged groups, although these factors were not significant when also accounting for age and gender.

Among the behavioural risk factors assessed, fast food consumption was most strongly associated with SSB consumption. Those who had consumed fast food in the past week had nearly twice the odds of being a consumer of SSBs and more frequent consumers of fast food (twice or more in past week) had over 5 times the odds. The linear relationship we observed between SSB consumption and other fast food consumption is consistent with other findings [13–17, 25, 26]. A qualitative study conducted with young adults in Australia identified strong social cues to purchase and consume SSBs [27]. This study found that SSB consumption was considered normal because of the ready availability, cheapness, and advertising and promotion of these drinks, and that SSB consumption was closely linked to purchasing fast-food and take-away meals. The strong association between fast food and SSB consumption is important because of compounding dietary risks from excess sugar, salt and fat. The pairing of SSBs with fast food is likely driven by availability at times of purchase, promotions, as well as pricing and ‘packaging’ of SSBs with food. Those who consumed juice were marginally more likely to have consumed fast food in the past week (bi-variate analysis only), and while 84% of those who consumed fast food twice or more per week also consumed SSBs, only 37% consumed 100% fruit juice.

We observed a clustering of ‘unhealthy’ behaviours (smoking and fast food consumption) with SSB consumption and not 100% fruit juice consumption, and an association between healthy behaviour (exercise) and 100% fruit juice consumption. Although juices frequently contain as much free sugar as soft drink (soda), community awareness of this is mixed, as we observed in our sample, and juice may have a ‘health halo’ not applied to soft drink [28, 29]. The relationship between exercise and different SSB types, e.g. sports drinks, was not investigated in this study; however, there was a positive association between exercise and consumption of 100% fruit juice, which persisted in the multivariate analysis. Given that some drinks are marketed as offering functional or health benefits, and the relationships we have observed in this study between health behaviours and juice consumption, consumer perceptions of different types of beverages high in free sugar (including juice) warrant further investigation.

This study found no relationship between self-reported weight status (BMI) and SSB consumption or 100% fruit juice consumption. Systematic reviews of prospective cohort and randomised control trial studies have clearly demonstrated that SSB consumption can lead to weight gain [2]. However, correlational studies are less consistent and the relationship tends to vary according to drink type and location. For example, one Australian study found that *soft drink* consumption was higher for those classified as either overweight or obese in South Australia but was only higher for those classified as obese in Western Australia [13]. Another Western Australian study found that those classified as overweight/obese were more likely to consume both sugar-sweetened and artificially sweetened soft drinks but there was no relationship for those who only consumed sugar-sweetened soft drinks [24]. BMI was not associated with SSB consumption but was associated with *fruit juice* consumption in a Norwegian study [30]. A US study of *sports and energy drinks* found that consumption was more likely for those classified as healthy weight [31]. It is important for future studies to assess drink types independently because a combined measure may mask important differences in the risk factors associated with consumption.

The results of this study suggested a lack of awareness of the contents of the drinks participants are consuming, as well as of the potential risks associated with excess consumption. Only 34% of respondents knew the approximate amount of sugar in a can of soft drink and a further one third underestimated the sugar content. While there was reasonable awareness of diabetes as a potential risk of excess SSB consumption among this sample (approx. two thirds of participants were aware), less than half recalled weight gain (42.5%), tooth decay (29.1%), or heart disease (14.9%) as potential risks. Frequent SSB consumers had lower rates of awareness of health risks and were more likely to underestimate sugar content in a can of soft drink than non-consumers. While the evidence of cardiovascular risk as a result of excess consumption is emergent, evidence for dental caries and weight gain is longer standing, highlighting the deficit in community understanding of the risks of excess SSB consumption. While one US study observed higher (70–80%) levels of awareness of weight gain, diabetes and dental caries [32] than that observed in the present study, these data reflected prompted awareness rather than unprompted, top-of-mind responses such as those assessed in this study. Several other US studies have also established poor awareness of the sugar content and calorie count of soft drinks [33, 34]. The results also indicate confusion about the relative merits of diet soft drinks compared to SSBs. Approximately one quarter of participants indicated diet drinks were less healthy than SSBs, a minority (17%) indicated they were healthier, and half indicated they were ‘about the same’. This consumer confusion is unsurprising given the changing state of evidence regarding diet beverages. Similarly to juice, consumers knowledge and beliefs about diet beverages warrant further investigation.

Industry repeatedly argues that information about sugar content and caloric count is available to consumer in nutrition information panels. While the US Food and Drug Administration has mandated the inclusion of added sugar on nutrition information labels in recognition of the scientific evidence about free sugars [35], information on added sugar content is not available to Australian consumers, despite advocacy for such a change. Furthermore, greater health literacy (i.e. capacity to understand basic health information needed to make appropriate health decisions) has been shown to be related to lower SSB intake [36]. This also highlights the need to either increase health literacy or provide information that is easy to understand, or both. There is a growing body of evidence that shows that that on-pack health warning labels [37–40] and mass media advertising on health effects of SSBs [41–43] help to improve understanding of the potentially harmful effects of consuming SSBs and may reduce SSB sales [44].

The present study analysed data from a representative face-to-face household survey in one Australian state and, while the results may not necessarily generalise to other states or countries, the results are consistent with those reported in other jurisdictions. The present study was cross-sectional so it is difficult to infer causality from the observed significant associations. Another limitation was the use of a brief, self-report consumption measure which relied on participants’ memory without additional prompting or cueing to aid recall. This may have produced an under-estimate of SSB consumption compared to an assessment using a 24-h recall interview method. It is possible that participants were not accurate in their self-reported body weight which may have reduced the likelihood of detecting an effect associated with BMI. It was not possible to compare responders to non-responders. However, an under-estimate of SSB consumption rates could have occurred through non-response bias if those with unhealthy lifestyles were less likely to respond to a health survey than those with healthy lifestyles.

## Conclusion

To conclude, the low rates of awareness of the health risks associated with SSB consumption and the low awareness of sugar content in SSBs, demonstrate that there is a need for greater consumer understanding. This is especially the case among frequent consumers who are the most at risk of harms associated with SSB consumption, and where there is also clustering with other unhealthy consumption behaviours. Potential strategies include public communication campaigns, the use of on-package warning labels which contain sugar content and/or risk information, and improvements to existing nutrition information panels so that quantity of ‘added sugar’ is clear. Further research that explores consumer response to risk information and perceptions of substitute beverages of fruit juice and diet drinks is warranted.

## Additional file

Additional file 1: Questionnaire and corresponding variable sub-categories. List of questions asked during the interview. (DOCX 21 kb)

## Abbreviations

BMI: Body Mass Index
SAHOS: South Australian Health Omnibus Survey
SSB: Sugar sweetened beverage
WHO: World Health Organisation
