# Supplementary material for: Who drinks sugar sweetened beverages and juice? An Australian population study of behaviour, awareness and attitudes
Source: BMC Obes. 2019 Jan 3;6:1. doi: 10.1186/s40608-018-0224-2 (PMC6317260; doi:10.1186/s40608-018-0224-2)
Supplement: Supplementary file 1 — Questionnaire and corresponding variable sub-categories. List of questions asked during the interview. (DOCX 21 kb) [file 40608_2018_224_MOESM1_ESM.docx]

Additional file 1: Questionnaire and corresponding variable sub-categories

| Question | Response | Variable sub-categories |
| --- | --- | --- |
| 1. Please record sex | *Male*  *Female* | Gender: male, female |
| 1. As health is often age related may I commence by asking your age? | *Record age* | Age (years): 15-24, 25-44, 45-64, 65 and over |
| 1. What is your height without shoes? | *Record Centimetres (OR) Feet/Inches*  *Don’t know* | BMI (calculated from Q3 and Q4): Underweight or healthy (≤25.0), Overweight (25.1-29.9), Obese (≥30), Don’t know either height or weight |
| 1. What is your weight (undressed in the morning)? | *Record Kilograms (OR) Stones/pounds*  *Don’t know* |  |
| 1. How many times in the last week have you eaten meals that were bought from fast food outlets like McDonalds, Hungry Jacks, Pizza Hut, KFC, Red Rooster, hamburger or fish and chip shops? | *Record number or (D) Don’t know/can’t say* | Fast food consumption (past week): none, once, two or more times |
| 1. During the past 7 days, on how many days did you drink a can, bottle or glass of a sugar-sweetened drink such as soft drinks (like coke/lemonade), energy drinks (like red bull), fruit drinks, sports drinks (like Gatorade) and cordial? This does not include diet or low joule drinks, or 100% fruit juice. | *Record 0-7 or (D) Don’t know/can’t say* | SSB consumption in past week (calculated from Q6 and Q7): none, any (1 or more), moderate (1-6 times), frequent (7+ times) |
| 1. On days that you did drink sugar-sweetened drinks, how many times per day did you usually drink them? Do not include diet/low joule soft drinks. | Once a day  Twice a day  3 times per day  4 or more times per day  Don’t know/can’t say  Refused |  |
| 1. During the past 7 days, on how many days did you drink a bottle, juice box or glass of 100% Fruit Juice? Note that this does not include fruit drink. | *Record 0-7 or (D) Don’t know/can’t say* | 100% fruit juice consumption (past week): none, one or more times |
| 1. Do you know of any illnesses or other health effects caused by drinking sweet drinks such as soft drinks (like coke/lemonade), energy drinks (like red bull), sports drinks (like Gatorade), or cordial? | *Multiple response. Prompt, anything else until no more responses* | Awareness of illnesses/health effects related to SSB consumption (yes/no): weight gain, diabetes, tooth decay, heart disease |
| 1. How many teaspoons of sugar do you think there might be in an average (375ml) can of soft drink like Coke, Fanta or Lemonade? | *Record number or (D) Don’t know/can’t say* | Teaspoons of sugar in can of soft drink: underestimate 0 to 7, approx. correct 8 to 12, overestimate 13 to 99, don’t know |
| 1. Compared to sugar sweetened soft drinks, do you think diet soft drinks are a more healthy choice, less healthy choice or about the same? | More healthy  Less healthy  The same  Don’t know  Refused | Diet soft drink versus SSBs: more healthy, less healthy, the same, don’t know |
| 1. Compared to sugar sweetened soft drinks, do you think 100% fruit juice has more sugar, less sugar or about the same amount? | More sugar  Less sugar  The same amount  Don’t know  Refused | 100% fruit juice versus SSBs: more sugar, less sugar, the same, don’t know |
| 1. Vigorous activity includes activities like fast cycling, jogging, doing an exercise class, and moderate activity includes things like brisk walking, medium paced swimming, walking to and from work, gardening, doing housework etc.   How many days in the past week have you done any vigorous physical activity for a total of at least 30 minutes, OR any combination of moderate and/or vigorous physical activity for a total of at least 60 minutes? This can include 60 minutes of moderate exercise only. | *Record days in the last week (0-7)*  *Don’t know*  *Refused* | Physical activity (past week): none, 1 to 6 days, everyday |
| 1. Do you currently smoke cigarettes, cigars, pipes or any other tobacco products? | Daily  At least weekly (not daily)  Less often than weekly  Not at all | Smoking status (calculated from Q14 and Q15): current smoker, ex-smoker, never smoked |
| 1. Over your lifetime would you have smoked at least 100 cigarettes or a similar amount of tobacco? | Yes  No |  |
| 1. Which of these groups best describes the highest qualification you have obtained? | Still at school  Left school at 15 years or less  Left school after age 15  Left school after age 15 but still studying  Trade qualification/ apprenticeship  Certificate/diploma – 1 yr full time or less  Certificate/diploma – more than 1 yr full time  Bachelor degree or higher | Highest qualification: High school or less, vocational, university |
| 1. *Record postcode* |  | Disadvantage quintile: Quintile 1 (most disadvantaged) to Quintile 5 (least disadvantaged)  Remoteness: Metropolitan, inner regional, outer regional, remote/very remote |
